# Green synthesis of nano-based drug delivery systems developed for hepatocellular carcinoma treatment: a review

**DOI:** 10.1007/s11033-023-08823-5

**Published:** 2023-10-10

**Authors:** Doaa S. R. Khafaga, Ahmed M. El-Khawaga, Rehab Abd Elfattah Mohammed, Heba K. Abdelhakim

**Affiliations:** 1 Department of Basic Medical Sciences, Faculty of Medicine, Galala University, Suez, 43511 Egypt; 2 Faculty of medicine for girls, Internal medicine Al Azher university, Cairo, Egypt; 3 Biochemistry Division, Faculty of Science, Cairo University, Giza, 12613 Egypt

**Keywords:** *Nanoparticles*, *Cancer*, *Hepatocellular carcinoma*, *Sorafenib*

## Abstract

This review presents an overview of one of the effective strategies for improving the anticancer impact of many drugs including sorafenib using a drug delivery system by employing nanoparticles that is produced through a biological system. The biological process has a lot of benefits, including being inexpensive and safe for the environment. Sorafenib is one of a multi-kinase inhibitor that inhibits molecularly targeted kinases. Because of its poor pharmacokinetic characteristics, such as fast elimination and limited water solubility, the bioavailability of Sorafenib is extremely low. More intelligent nano formulations of sorafenib have been developed to boost both the drug’s target ability and bioavailability. Researchers in a wide variety of sectors, including nanomedicine, have recently been interested in the topic of nanotechnology. It is possible for the body to develop resistance to widely used drugs available for treatment of liver cancer, including sorafenib. As a result, our goal of this research is to highlight the efficacy of nanomedicine-based drug delivery system to enhance drug’s cancer-fighting properties. Because of their magnetic properties, certain nanoparticle materials can be employed as a carrier for the medicine to the exact place where the cancer is located. This can lower the amount of the drug that is administered with no impact on the normal cells.

**Supplementary Information:**

The online version contains supplementary material available at 10.1007/s11033-023-08823-5.

## Introduction

Cancer is one of the leading causes of death. It develops through a multistep process called carcinogenesis that involves many cellular physiological systems like cell signaling and apoptosis [1]. Hepatocellular carcinoma is one of the most aggressive malignancies, ranking as the third greatest cause of cancer-related mortality .It is the fifth most prevalent malignancy in males and the seventh most common in women [2]. The only systemic treatment for hepatocellular carcinoma that is widely accessible is sorafenib. It is an orally active multi target kinase inhibitor that targets intracellular serine-threonine kinases Raf-1 and B-Raf as well as several cell surface tyrosine kinases (such as VEGFR-1, -2, -3, and PDGFR-). As a result, sorafenib reduces angiogenesis and tumor cell growth while promoting tumor cell death [3]. Sorafenib has adverse effects that restrict its usage, similar to those of many other chemotherapeutic drugs, including toxicity, hypertension, mucositis, alopecia, and hand-foot skin response [4]. There are several drug delivery and targeting systems being developed right now. Synthetic polymers, microcapsules, liposomes, and many other drug delivery systems all have the trait of enhancing drug bioavailability and enhancing drug accumulation at the target site. This is crucial for anticancer drugs, which should ideally be given locally and have little to no impact on healthy tissue [5]. The controlled release characteristics of the nanosystems, also enables decrease in treatment dosage and frequency of the drugs. Drug molecules are not degraded, and undesirable toxicities associated with anticancer drugs can be reduced by nanoparticulate drug delivery methods. By overcoming all the biological and physical barriers that often prevent conventional drugs from working, nanoparticles can show successful effect in treating cancer. Zinc oxide nanoparticles (ZnO NPs) is metal nanoparticles that are commonly employed in sunscreens and antibacterial medicines, they are also cholesterol biosensors and dietary modulators for regulating diabetes and hyperlipidemia [6]. Superparamagnetic iron oxides (SPIONs) are small crystals of iron oxide (typically magnetite Fe_3_O_4_ or maghemite Fe_2_O_3_) with surfaces modified to improve colloidal stability in aqueous environments. The SPION-based drug - delivery system must meet certain parameters to find success. The coating or carrier should make the delivery system hydrophilic enough so that it can be easily dispersed in water. In addition to this, it should provide functional groups that lead to subsequent modification to control the release of the drug or to bind targeted units. Nanosystems based on silver nanoparticles have been investigated for their potential use as carriers for a wide range of medicinal compounds, such as those with anti-inflammatory, antioxidant, antibacterial, and anticancer properties [7]. Bee venom, a natural substance that has been utilized as a traditional medicine for the treatment of hepatocellular carcinoma [8].

## Nanomedicine

**Fig. 1:**
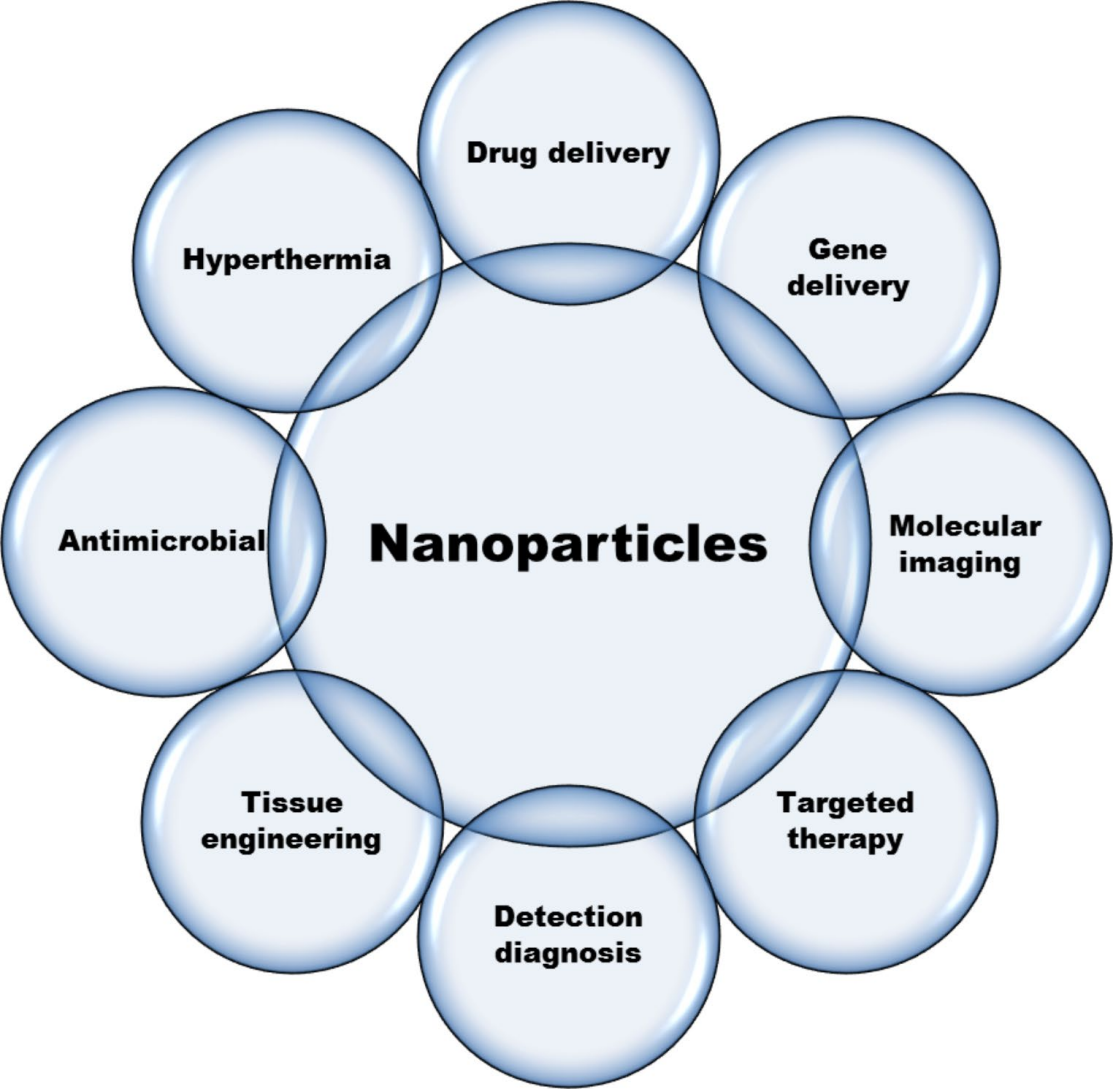
Biomedical applications of nanoparticles

The prefix “nano” refers to extremely tiny size. It involves combining atoms, molecules, and compounds to create structures with particular properties. It deals with substances with sizes ranging from 0.1 to 100 nm [9]. The physical, chemical, and biological systems are all addressed by nanotechnology. Nanotechnology research and technology offers evolution in materials and manufacturing, nanoelectronics, medicine healthcare, energy, biotechnology, information technology, and national security [10]. Nanomedicine is a growing discipline that uses nanosized materials for illness detection and treatment. Drugs that are poorly absorbed are encapsulated with nanomaterials or vehicles for controlled and prolonged drug release [11]. Enhanced targeted selectivity and improved delivery efficiency are the two main aims in the development of therapeutic medicines. To achieve these goals, therapeutic drugs should be administered to the cancer without damaging normal tissues. Nanoscale devices can be coupled with tumor-specific ligands, antibodies, anticancer drugs, and imaging probes. Due to the fact that the size of these nano-devices is comparable to that of a single cancer cell or even smaller, these molecules connect with tumor-specific proteins on the surface and inside of cancer cells and are easily delivered through blood vessels that are leaking. Figure 1 demonstrates some of applications of nanoparticles in biomedical fields. As a result, their use as cancer cell-specific delivery vehicles will represent a considerable improvement over what is now available for cancer therapies and imaging. Cancer nanotechnology has the prospect of overcoming some of the present barriers to cancer therapy [12].

### Bio/Green synthesis of nanoparticles

Several techniques can be generated for the synthesis of NPs; these methods are generally classified as (1) bottom-up and (2) top-down approaches.

The top-down synthesis process employs a destructive technique which starts with a breaking down a bigger molecule into smaller components that are transformed into nanoparticles. Physical vapor deposition (PVD), Grinding/milling are examples of this technology. Conversely, the bottom-up strategy operates in reverse, because of the NPs are created from simpler ingredients; hence, this approach is also referred to as the building up approach. Green synthesis and sol gel are examples of this category [13].

**Fig. 2:**
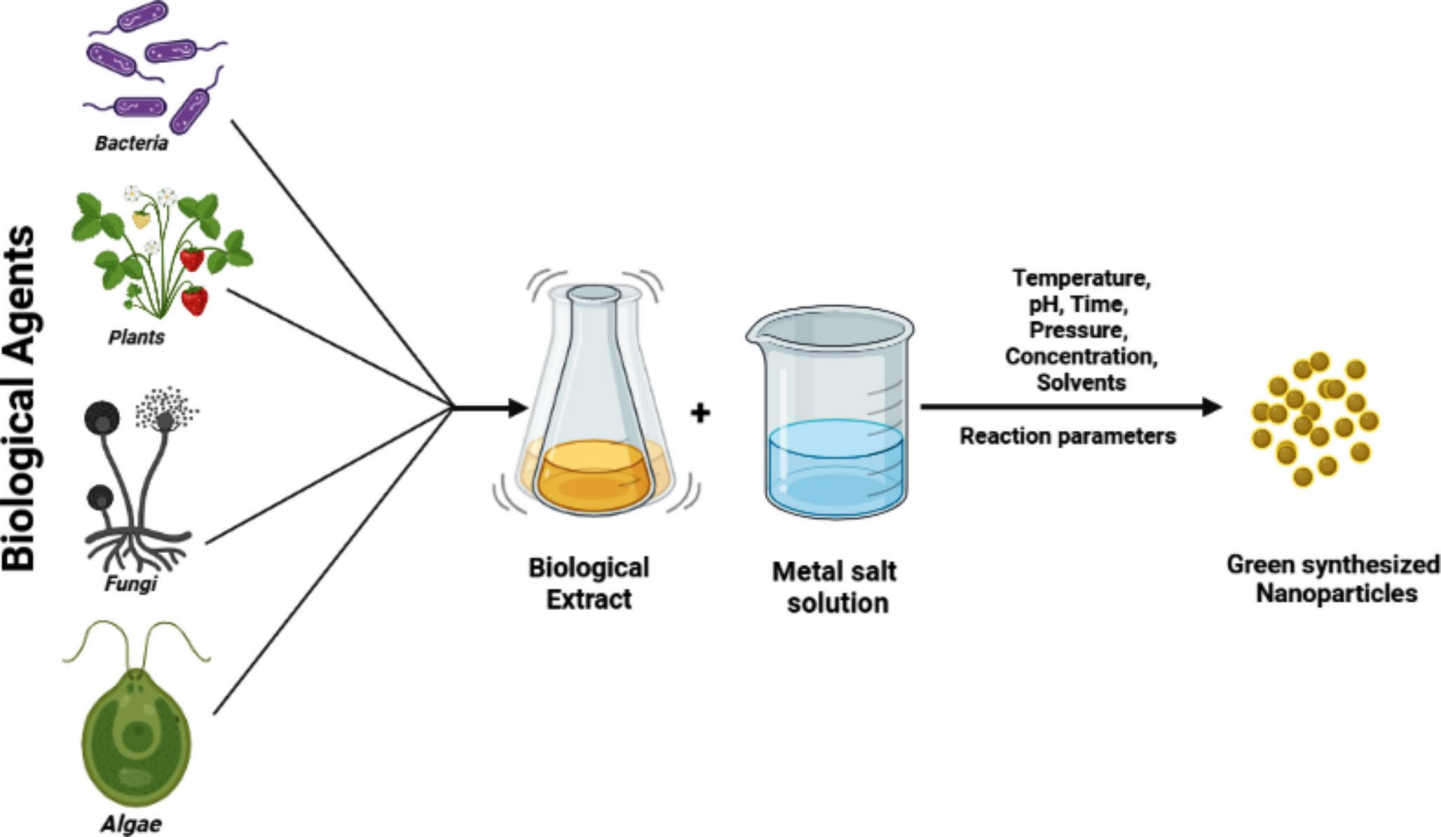
Schematic figure demonstrates the green synthesis methods of nanoparticles

The green synthesis approach of nanoparticles is unique and it is less time intensive. The synthesis of nanoparticles (NPs) through green methods can be achieved by utilizing a diverse range of resources, including plants and plant-derived materials, algae, fungi, yeast, bacteria, and viruses as illustrated in Fig. 2. It can be used in various industries, including agriculture, medical and electronic industries [14]. Thus, it is expected that synthesis of nanoparticles using biological principles would be simple, cost- effective, safe, and eco-friendly. Generation of nanoparticles through exploiting plants acquired significant interest, primarily due to its simple technique and it does not involve intricate process like maintenance of microbial culture and many purification steps [15].

### Nanoparticles used in drug delivery system

#### Inorganic nanoparticles

**Fig. 3:**
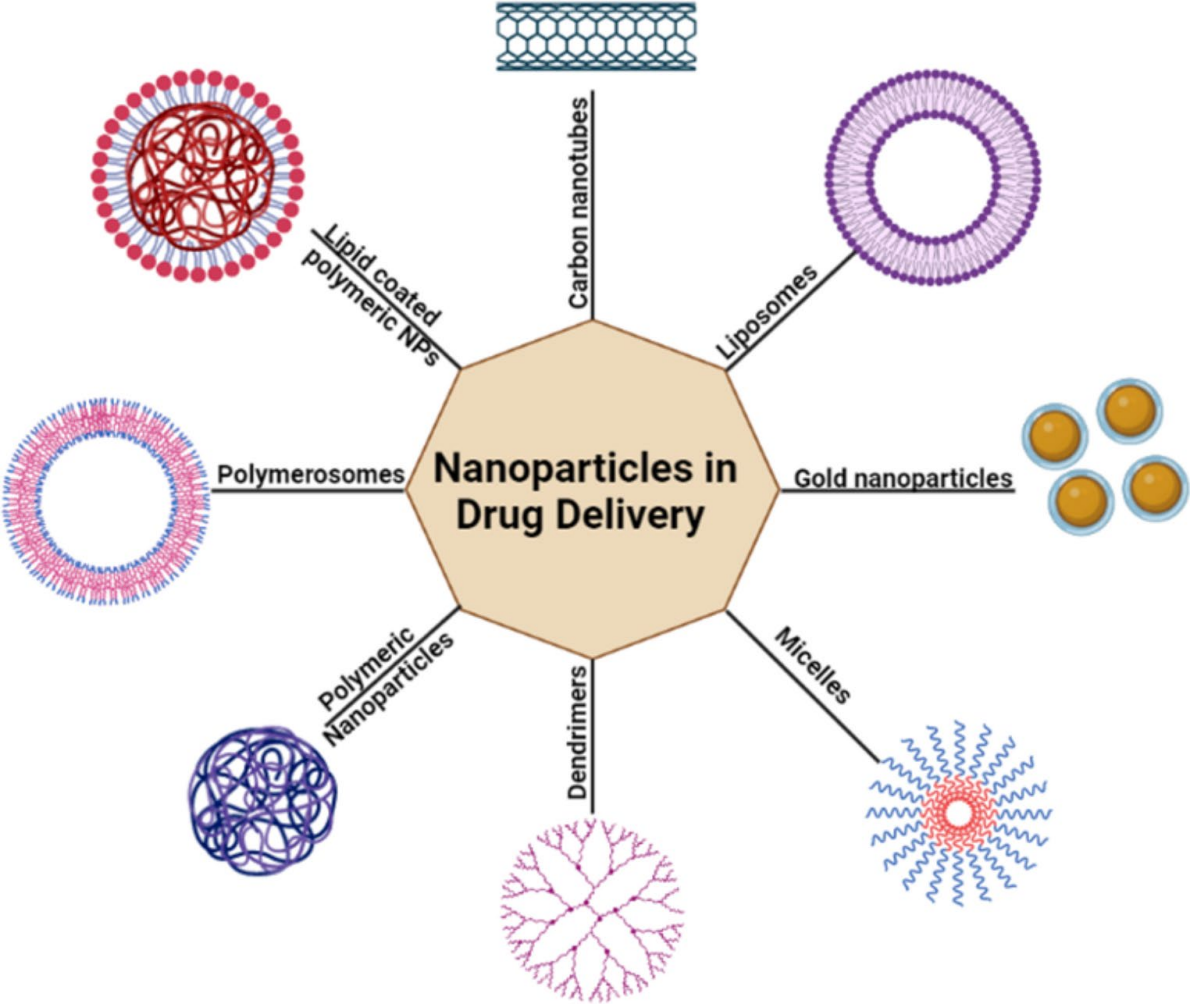
Nanoparticles used in drug delivery in cancer

Although there are no many studies on inorganic nanoparticles including those made of iron oxide, silver, gold, and silica compared to other nanoparticle, only few of them have been approved for use in therapy as listed in Table 1. The rest are still being tested in clinical trials. Metal nanoparticles as silver and gold have unique property known as surface plasmon resonance (SPR), which is not found in liposomes, dendrimers, and micelles. This character displayed several benefits, including great biocompatibility and surface functionalization versatility. Although two mechanisms, paracellular transport and transcytosis, have been hypothesized for their uptake and in vivo transport studies focused on their drug delivery-related activity were unable to establish whether the ionized or particulate form is particularly linked to their toxicity [16]. Cancer drugs can be binding to the surfaces of gold nanoparticles (AuNPs) via ionic or covalent bonding as well a physical absorption. These drugs can be then regulated and administered using biological or light stimulation [17]. However silver nanoparticles have antibacterial properties, there has been little research into their potential utility in medicine delivery. Prusty and Swain created a hybrid system polyacrylamide/dextran nano-hydrogels covalently connected with silver nanoparticles that had an in vitro release of 98.5% for ornidazole [18]. Figure 3 demonstrates different types of nanoparticles used as anticancer agents.

#### Metallic nanoparticles

In recent years, metallic nanoparticles have seen increased use in various medical applications, such as drug delivery, biosensors, and photoablation treatment. Metallic nanoparticles offer several advantages including low cost, ease of synthesis, and the flexibility to control the form and size of the NPs. On the other hand, Metallic nanoparticles also have some disadvantages. One of the main limitations is that macrophages of the phagocytic system are capable of rapidly eliminating them even before they reach the site of cancer cells. Another limitation associated with the use of oxide nanoparticles in active substance transport systems is the possibility of aggregation of these materials, resulting in a significant increase in particle size. In addition, the modification and functionalization of these nanoparticles with specific functional groups allow them to bind to antibodies, drugs and other ligands, become these making these systems more promising in biomedical application [19]. Although gold, silver, iron, and copper are the most thoroughly researched metallic nanoparticles, several examples of metallic nanoparticles, such as titanium oxide, zinc oxide, platinum, selenium, palladium, gadolinium, and cerium dioxide, are receiving increased attention [20].

#### Nanocrystals

Milling, high pressure homogenization, and precipitation are the most often used techniques for producing nanocrystals. Thus, nanocrystals offer several advantages and disadvantages. Among the advantages, there are; higher solubility, faster dissolution rate than conventional particles, potential for passive and active targeting of drugs and reduced tissue irritation in case of subcutaneous/intramuscular administration. Fewer are the disadvantages, such as; Uniform and accurate dose cannot be achieved, Physicochemical-related stability problems and Bulking sufficient care must be taken during handling and transport [21]. Using chitosan microparticles encapsulating cinaciguat nanocrystals is another example of using nanocrystals for hydrophobic drug delivery to the lungs. The nanoparticles were created for sustained drug delivery by utilizing the polymer’s swelling and muco-adhesive capabilities. More study is required to confirm the efficacy of these approaches in the drug delivery systems [22].

#### Biopolymeric nanoparticles

Several various types of biopolymeric polymers are employed in drug delivery systems. Many of these materials and their characteristics will be highlighted in the following.

**a- Chitosan**.

Chitosan-based nanoparticles are commonly used for sustained drug delivery systems. Silva et al. created an isotonic 0.75% w/w hydroxypropyl methylcellulose (HPMC) solution incorporating chitosan/sodium tripolyphosphate/hyaluronic acid nanoparticles to transport the ceftazidime antibiotic to the eye [23]. Pistone et al. created chitosan pectin, and alginate nanoparticles as prospective options for drug delivery into the buccal cavity. The solubility of the nanoparticles in saliva was used to determine the biocompatibility of the formulations, and their cytotoxicity was calculated using the oral cell line [24].

**b- Alginate**.

Alginate is another biopolymeric substance that has been exploited in drug delivery. Alginate contains carboxyl groups in each residue, classifying it as an anionic mucoadhesive polymer with higher mucoadhesive compared to cationic and neutral polymers. In diabetic rats, Patil and Devarajan used nicotinamide to permeate insulin-containing alginate nanoparticles to lower glucose and increase insulin levels. Sublingual nanoparticles (5 IU/kg) with nicotinamide had 100% pharmacology and 80% bioavailability. Chitosan-coated alginate nanoparticles were produced by Costa et al. to increase daptomycin absorption into the ocular epithelium and accomplish antibacterial effects. Ocular epithelial cell culture models were employed to measure in vitro permeability [25].

**c- Xanthan gum**.

Menzel et al. investigated a new nasal release excipient. Cys-MNA, a primary polymer, was linked to xanthan gum. The conjugate was tested for binder amount, muco-adhesiveness, and degradation resistance. Polymer grammes got 252.52 20.54 mol of binder. Grafted polymer muco-adhesion was 1.7 times higher than thiolated xanthan and 2.5 times that of native. When the polymer was withdrawn from the mucosa, nasal epithelial cell ciliary beating was unchanged and only reversible [26].

**d- Cellulose**.

Cellulose as well as its derivatives are commonly employed in drug delivery systems to modulate drug release by altering drug solubility and gelation [27]. Elseoud et al. [28] investigated repaglinide oral release using chitosan nanoparticles and cellulose nanocrystals. Chitosan nanoparticles had a mean size distribution of 197 nanometers, while hybrid nanoparticles of chitosan and cellulose nanocrystals containing RPG had a mean size distribution of 198 nanometers. Chitosan hybrid nanoparticles and RPG-containing oxidized cellulose nanocrystals had 251–310 nm mean diameters. Hydrogen interactions between the drug and the cellulose nanocrystals extended the drug release, resulting in lower drug release in nanoparticles created with oxidized cellulose nanocrystals [28].

#### Liposomes

Alec Bangham discovered the liposomes in 1960. They are widely employed in the cosmetics and pharmaceutical industries for the transportation of various compounds and they are one of the common researched drug delivery carriers. They are a well-established method for improving drug delivery. They are spherical vesicles composed of steroids and phospholipids that range in size from 50 to 450 nm. They are thought to be superior drug delivery transporters because if similarity of their membrane structure to cell membranes and easier drug integration [29]. They also stabilize medicinal molecules, promote biodistribution, work with hydrophobic and hydrophilic drugs, and they are biodegradable and biocompatible. Four liposome types: (1) Conventional liposomes: A lipid bilayer surrounds an aqueous core and produces cationic, anionic or phospholipids and neutral cholesterol. Hydrophobic or hydrophilic components can fill the aqueous space and lipid bilayer. (2) PEGylated types: PEG is added to the liposome surface to create steric equilibrium. (3) ligand-targeting: antibodies, polysaccharides, and peptides are attached to the surface of the liposome’s e or PEG chain ends. (4) The ranostic liposomes: a nanoparticle, targeting, imaging, and therapeutic element make up this type of liposome [30]. Liposomes in the bloodstream interact with opsonins, HDLs, and LDLs. Immunoglobulins and fibronectin help RES to recognize and remove liposomes. Liposomes destabilize HDLs and LDLs. Liposomes gather more in organs like the liver and spleen, which might help alleviate pathogenic diseases, but it may impede the clearance of lipophilic anticancer drugs in tumors [31].

#### Polymeric micelles

Polymeric micelles are nanosized structures constructed of amphiphilic block copolymers that cluster in aqueous solution to form a core shell shape. The hydrophobic core can be incorporated with hydrophobic medicines (for example, camptothecin, docetaxel, paclitaxel), while the hydrophilic shell renders the entire system water soluble leading to stabilization of the core. Polymeric micelles are typically less than 100 nm in size and have a limited distribution to hinder rapid renal elimination, this enable them to accumulate in tumor cells via the EPR effect. Furthermore, the polymeric shell prevents nonspecific reactions with biological compounds. These nanostructures have a high potential for hydrophobic drug delivery because their internal core structure that improver drug absorption, resulting in increased stability and bioavailability [32]. Polymeric micelles was found to be useful for delivery of both cancer drugs and ophthalmic drugs [33]. Li et al. encapsulated dasatinib in 55 nm nanoparticles generated by micellation of PEG-b-PC for treatment of proliferative vitreoretinopathy (PVR). The nanoparticles were noncytotoxic to ARPE-19 cells and had a limited distribution [34].

#### Dendrimers

Dendrimers are well-defined, monodisperse, strongly bifurcated three-dimensional structures. Because of their spherical shape and their ease of controlled functionalization, they would be excellent drug delivery agents [35]. Dendrimers may be produced using two methods: The first is a divergent path which starts from the core and subsequently extends outwards, while the second is a convergent route that begins from the dendrimer’s exterior [36]. There are difficulties to dendrimers’ application in medicine due to their amine groups. Due to the toxicity of these positively charged or cationic groups, dendrimers are commonly modified to minimize these groups. Dendrimers can be loaded with drugs through simple encapsulation, electrostatic interaction, or covalent conjugation [37]. The advantages and disadvantages of different nanocarriers types are listed in table S1.

### Nano based drug delivery systems

The newest technology in the field of nanoparticles is designing drugs at the nanoscale, which has benefits such as the ability to change properties like drug release profiles, diffusivity, bioavailability, immunogenicity, and solubility. This can improve and simplify administration of a drug, reduce its toxicity, minimize its side effects, increase its bioavailability, and lengthen its shelf life [38]. The newly designed drug delivery systems are either targeting a specific place or made so that therapeutic substances are released slowly at that place. Their development includes self-assembly, in which building parts come together on their own to form well-defined shapes or pattern. Nanostructures can autonomously or passively deliver drugs. Hydrophobic effect helps drugs enter the structure’s inner cavity. Because of the low pharmaceutical concentration in a hydrophobic environment, nanostructure materials tailored to specific locations release the right amount of drug [39]. For easy delivery, drugs are directly incorporated to the carrier nanostructure material. Since the drug does not reach the target location and soon detached from the carrier, its bioactivity and effectiveness are reduced if it is not liberated from its nanocarrier system at the specific time [40]. Table S2 demonstrates the loading of some synthetic drug on different nanocomposites for cancer treatment.

### Drug delivery systems based on natural products

Natural compounds as mentioned in Table 1, often known as natural products, are chemical ingredients that may be found in plants and microbes. Throughout history, natural ingredients have been employed as herbal treatments. Many Bioactive compounds which are mostly specific secondary metabolites with antioxidant, inflammatory, immune modulative potential, antimicrobial properties, etc. are derived from natural sources, but large pharmaceutical companies have not paid them enough attention. This could be related to the outdated belief that the only practical application for natural compounds is as antibiotics. After World War II, natural products were very successful as antibiotics, so the two ideas have become interchangeable [41]. The largest issue with using natural products to treat the cancer is that they aren’t very bioavailable. For example, people who took curcumin by mouth needed 3.6 g/day to get blood levels of 11.1 nmol/L. Curcumin levels in the blood could not be found in those who got smaller doses [42].

Some natural substances have biological activity and can be utilized to treat human diseases. Natural substances have been explored and utilized in complementary and alternative medicine for the treatment of cancer, infectious illness, and other diseases as listed in Table 3. By enhancing bioavailability, nanoparticles can increase the efficacy of natural chemicals in disease therapy and prevention. Curcumin, resveratrol, and EGCG, are extremely lipophilic. Because highly lipophilic chemicals do not dissolve efficiently in the circulation, they are not suitable for drug delivery. Because these compounds have a limited bioavailability, significant amounts of them must be taken to obtain the required therapeutic effects. These drugs’ high dosage sizes can cause acute toxicity or poor patient compliance. Simply encapsulating these highly lipophilic molecules increases their water solubility and efficiency [43].

**Table 1:** Different natural products loaded nanoparticles and their purpose

Natural compound	Loaded nanoparticles	Purpose	Ref.
Gallic acid	PLGA	Treat Acanthamoeba triangularis	[44]
Curcumin	Convert to nano-curcumin	-Increase HDL levels -Decrease inflammatory markers -Improve the glycemic profile with cardioprotective effects	[45]
Green tea	poly (lactide-co-glycolic acid) (PLGA)	-Response toward inflammation in human dermal fibroblasts	[45]
Berberine	Nanoemulsions (NEs)	-High-fat diet and streptozocin-induced diabetic mice	[46]
Baicalin	Nanostructured lipid carriers	-Lowered blood glucose	[46]
Dietary curcumin / resveratrol (RSV)	DSPE-PEG_2000_-SP94	-Treatment of hepatocellular carcinoma	[47]
Silibinin Metformin	PLGA-PEG	-Improves chemotherapy in human non-small cell lung cancer A549 cells	[48]
Curcumin	PLGA	-Anti-Gastric Cancer -Anti-Helicobacter Pylori Effect	[49]
Lawsone	Niosome	-Antitumor activity in MCF-7 breast Cancer cell line	[50]
Ganoderic acid	Nano-lipidic	-Improvise treatment of hepatocellular carcinoma	[51]
Berberine	Mesoporous silica	-Liver cancer therapy	[52]

### Targeting of drugs

Drug targeting can be active or passive when using nanomaterials or nano formulations as drug delivery methods. Active targeting involves coupling components such as antibodies and peptides with a drug delivery system to receptor structures expressed in the target location. The produced drug carrier complex circulates through the circulation and is driven to the target site by affinity or binding influenced by variables such as pH, temperature, molecular site, and shape in passive targeting. Cell membrane receptors, lipid membrane components, and cell surface antigens or proteins are the primary targets of the body [40]. Drug delivery systems enhanced by nanotechnology are now being developed with the aim of treating and preventing cancer.

### Drug delivery process and mechanism

**Fig. 4:**
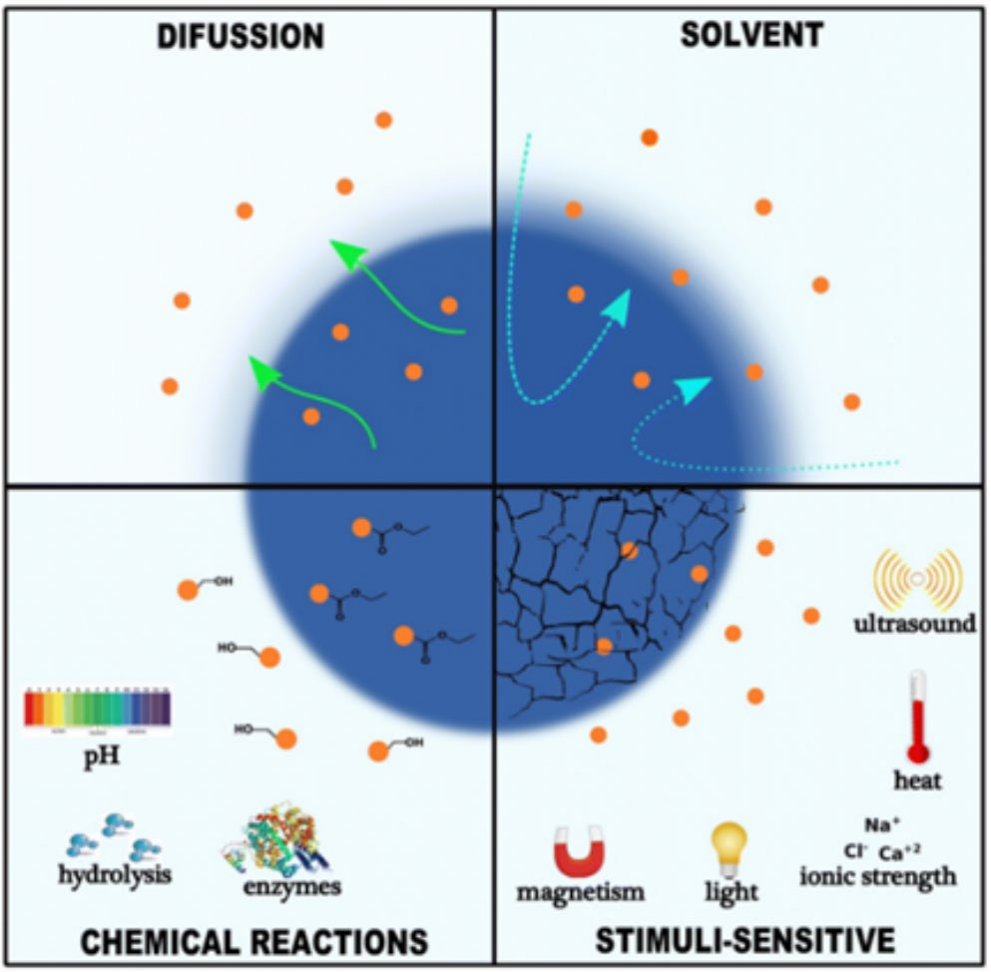
Controlled release mechanisms of drugs by different nanocarriers, Reprinted with permission from Ref. [40] Copyright © 2018 Journal of Nanobiotechnology

With the development of nanomedicine, drug discovery/design, and drug delivery systems, numerous therapeutic methods and conventional clinical diagnostic methodologies have been studied to improve diagnostic precision and drug specificity. For instance, novel pharmaceutical delivery systems are being researched with a focus on ensuring localized action, reducing toxicity, and increasing bioavailability in the body [53]. From this view, it appears that drug design is a possible way to find innovative lead drug using data about biological targets. The development and growth of the sector depends on improvements in computer science and the improvement of experimental techniques for classifying and purifying proteins, peptides, and biological targets [54]. Numerous researches have been published in this topic; they focus on the rational design of various compounds and highlight the need to investigate alternative drug release mechanisms as shown in Fig. 4.

Despite the fact that various nanocarriers have distinct drug release rates, techniques are now being developed to increase the selectivity of the nanostructures to target parts of the body [55], and to decrease immunogenicity by chemical functionalization or coating with a variety of compounds, such as polysaccharides [56] peptides [57], etc.

## Future of drug delivery system based on nanomaterials

One of the most fascinating academic disciplines right now is nanomedicine. Over the past two decades, scientists have poured resources into studying this topic, leading to the publication of 1500 patents and the finalization of large numbers of clinical investigations [58]. As mentioned above, cancer stands out as an excellent example of a disease where nonmedical technologies have helped in both screening and treatment. By using the a wide variety of nanoparticles to deliver the exact amount of drug to the affected cells, like cancer/tumor cells, without disrupting the physiology of the normal cells, the field of nanomedicine and nano-drug delivery systems has established itself as a trend that will continue to dominate future research and development for many years to come [40].

## Hepatocellular carcinoma

Hepatocellular carcinoma is the most prominent kind of liver cancer (HCC) [59]. Targeted treatment is emerging as a very promising method. Wherein a drug detects and selectively attaches to the target, causing cancer cells to die while having little effect on normal liver tissue [60]. Its targeted therapeutic techniques entail the suppression of certain growth factor receptors and the signaling cascades that they control [61].

### **Treatment of hepatocellular carcinoma**

**Fig. 5:**
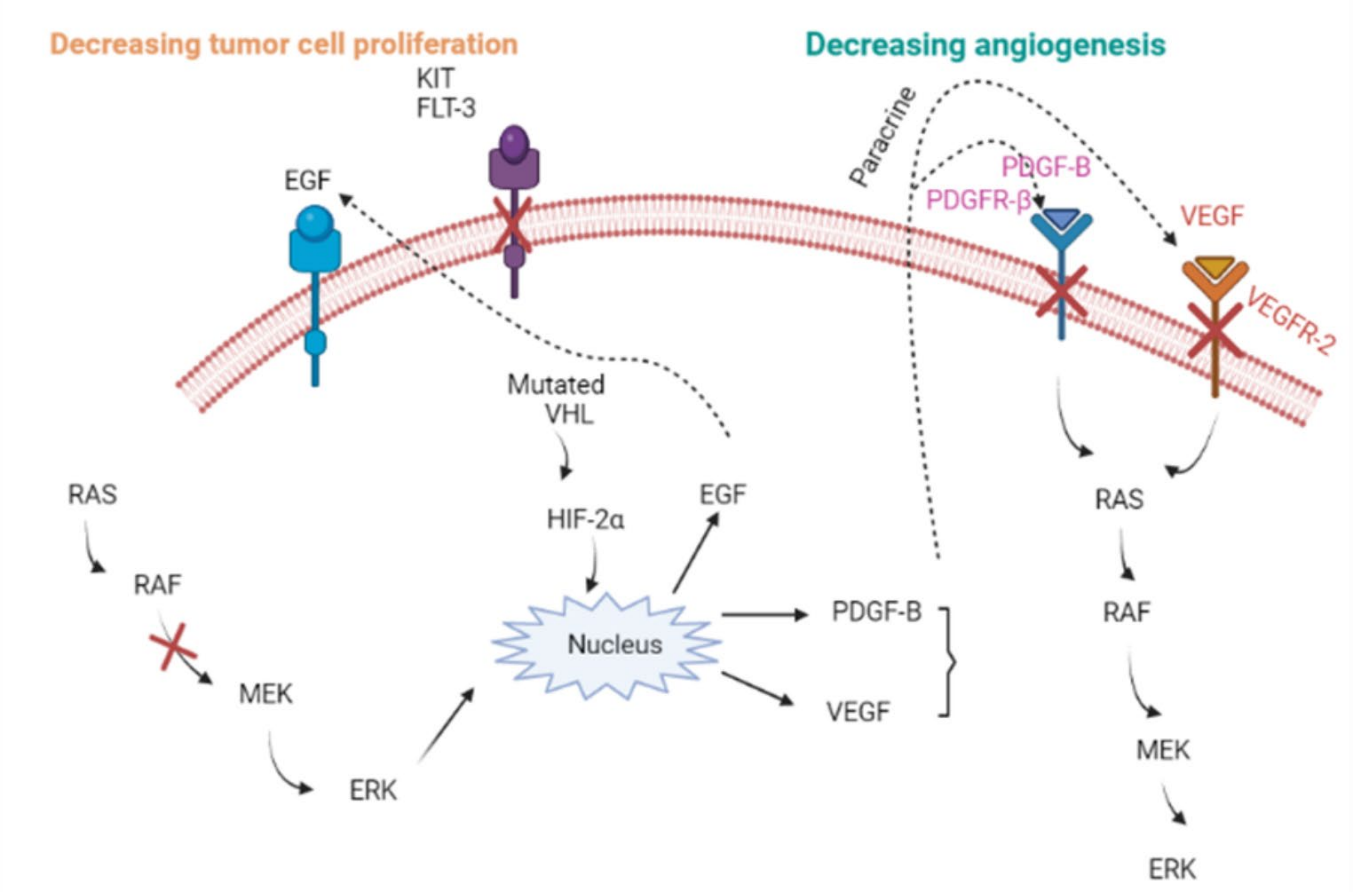
Multi-kinase inhibition (MKI) mechanism by sorafenib

The treatment of HCC is possible through a variety of approaches, including **(1)** oncolytic virus treatment, which uses a naturally existing virus to recruit the immune system to combat malignant cells and potentially eliminates cancer cells without damaging normal tissues. Oncolytic viruses enter hepatocellular carcinoma (HCC) cells, proliferate within them, and then release second-generation viruses that infect the surrounding HCC cells. Immune cells (innate and adaptive immune cells) are recruited in response to cytokine production, which ultimately results in immune clearance and HCC apoptosis [62]. **(2)** The immune system plays a crucial role in the war against cancer. The treatment of cancer has been greatly improved by immunotherapy. Checkpoint inhibitors for the programmed cell death protein 1 (PD-1/PD-L1) and cytotoxic T-lymphocyte-associated protein 4 (CTLA-4) pathways have been developed for the treatment of cancer. The first CTLA-4 inhibitor was licensed by the FDA in 2011, and its use has led to a dramatic increase in survival rates for patients with metastatic melanoma [63]. **(3)** Surgical treatments are conducted under imaging control, with maximum effectiveness for nodules smaller than 3 cm in diameter, with an 80% full response rate. However, several recurrences have been documented using such procedures [64]. **(4)** Liver transplantation is the final treatment option for hepatocellular carcinoma (HCC), which allows treatment of the underlying liver diseases in addition to the cure of the HCC. However, because of the extremely strict conditions for liver transplantation in HCC patients (known as the Milan criteria, which include patients with one tumor of less than 5 cm or up to three tumors of less than 3 cm), only a very small percentage of patients can get a liver. Patients who satisfied the Milan criteria had a 75% chance of surviving their condition [65]. However, the danger of reinfection exists after a liver transplant, and nearly half of patients develop post-transplant liver cirrhosis [64]. Sorafenib is an example of a drug used in chemotherapy. Sorafenib decrease the activity of Raf-1, B-Raf, and kinases in the Ras/Raf/MEK/ERK signalling cascade to suppress cancer cell growth. Sorafenib inhibits angiogenesis by targeting hepatocyte factor receptor (c-Kit), Fms-like tyrosine kinase (FLT-3), vascular endothelial growth factor receptor (VEGFR)-2, VEGFR-3, platelet-derived growth factor receptor (PDGFR-), and other tyrosine kinases as illustrated in Fig. 5 [66]. Sorafenib has also been shown in preclinical tests to be effective in a variety of tumor cells, including breast cancer MDA-MB-231 (with G463V b-raf and k-ras gene mutations), melanoma LOX, and pancreatic BxPC3 cells, as well as colon cancer HCT116, DLD-1, and Colo-205 cells [67] and other tumor cell lines.

Sorafenib is approved for the treatment of hepatocellular carcinoma, renal cell carcinoma, and thyroid cancer. Its anti­tumor actions may include reduction of tumor growth and development, blockage of metastasis and angiogenesis, and down regulation of systems that protect tumors from apoptosis [68]. The therapeutic efficacy of sorafenib, specifically in the treatment of renal cell carcinoma, is attributed to its ability to suppress angiogenesis, a mechanism shared by other antiangiogenic medications. The investigation of sorafenib’s role in treating advanced breast cancer was conducted due to the proven efficacy of inhibiting angiogenesis in this condition [69]. Sorafenib has serious side effects, including gastrointestinal symptoms, constitutional, diarrhea, weight loss, and dermatologic manifestations. It can cause hypertension, and even termination of therapy can occur due to severe side effects, and some individuals are initially resistant to sorafenib [70]. Recent research suggests that epigenetics, transport pathways, controlled cell death, as well as the initiation and development of sorafenib resistance in HCC may be influenced by the tumor microenvironment [71].

## Sorafenib resistance in HCC

Sorafenib resistance is caused by ATP binding box (ABC) transporters, which diminish chemotherapy efficiency by drawing medicines out of cancer cells and significantly impact anticancer therapy results. Exosomes are also intercellular information carriers and tumor microenvironment regulators. Exosomes eliminate toxic macromolecules from normal cells, but cancer cells may take over this process. For example, cancer cells resistant to drugs can be put in exosomes and moved out of tumor cells. Below, we talk more about the mechanisms of sorafenib resistance in HCC [72].

### ABC transporters

ABC transporters have been seen to interact with sorafenib and other TKIs. TKIs may act as substrates or inhibitors depending on pump expression, drug type, transporter affinity, and concentration. TKIs as ABC transporter antagonists increase anticancer treatment options and clinical drug resistance strategies. Di Giacomo et al. [73] Investigations were made on CRYO’s potential to block ABC pumps and enhance HCC cells’ response to sorafenib at safe dosages. CRYO is a naturally occurring sesquiterpene component of many essential oils. By upregulating MRP1 and MRP2, they were able to isolate a clonal subgroup of human HCC cells with higher multidrug resistance (MDR). Moreover, CRYO promoted sorafenib’s intracellular accumulation, boosted its cytotoxic response, and inhibited its degradation. In HepG2/S HCC cells, COP9 signaling corset 5 (CSN5) was linked to sorafenib resistance, according to another study. After CSN5 was silenced, sorafenib resistance was overcome, and several proteins linked to resistance (such as ABCB1, ABCC2, and ABCG2) were repressed. In addition, SR cells expressed higher ABCC1-3. enhanced SR cell invasion and migration, as well as a rise in the proportion of CD44^+^ to CD44^+^ CD133^+^ cells in SR cells [71].

### Exosomes

**Fig. 6:**
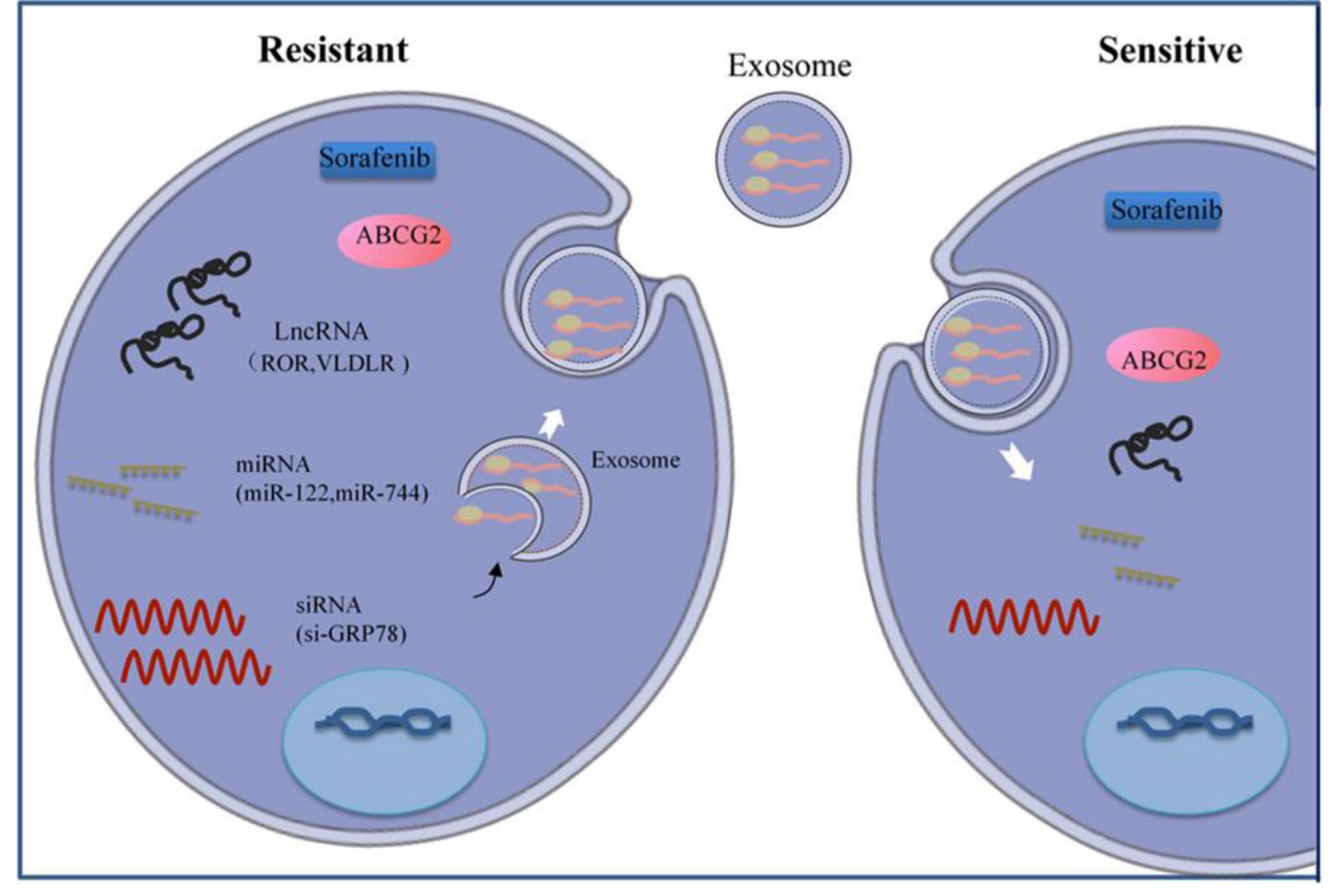
Exosomes used to deliver mRNA, lncRNAs, miRNAs, and siRNAs s for HCC treatment. Reprinted with permission from Ref. [71] Copyright © 2020 Journal of Signal Transduction and Targeted Therapy

Exosomes, which are small extracellular vesicles (EVs), have become a therapeutic target because they allow cells to communicate with one another. LincRNA-VLDLR (linc-VLDLR) was shown to be significantly overexpressed in malignant liver cells. The expression of lincVLDLR in cells and EVs produced from HCC cells increased after exposure to several anticancer treatments (e.g., sorafenib). Incubating recipient cells with EVs reduced chemotherapy-induced cell death while increasing linc-VLDLR expression. Also, linc-VLDLR knockdown decreased the expression of ABCG2 (ATP binding cassette, subfamily G member 2), and the effect of linc-VLDLR knockdown on sorafenib-induced cell death was lessened by overexpressing this protein. MiRNAs, like lncRNAs, can be carried in exosomes. It has been shown that miR-122-transfected adipose tissue mesenchymal stem cells (AMSCs) efficiently package miR-122 in secreted exosomes. This is likely how AMSCs and HCC cells communicate about miR-122, which makes HCC cells sensitive to sorafenib by changing the expression of miR-122 target genes. When miR122-exo was injected into the tumor, sorafenib worked much better against HCC tumors in living animals. Li et al. [74] Sorafenib and si-GRP78-modified exosomes may block HCC cells from spreading and invading by targeting GRP78. Si-GRP78-altered exosomes can resensitize sorafenib-resistant cancer cells. Exosomes from dendritic cells (DCs), which are crucial for primary and secondary immune responses, may treat some malignancies. Exosomes were loaded with certain molecules such functional proteins, ncRNAs, and chemotherapeutic medicines as represented in (Fig. 6). Future treatment approaches for advanced-stage HCC must take into account the resistance mechanisms including exosome-mediated crosstalk [71].

## Enhancement of sorafenib using drug delivery system

**Fig. 7:**
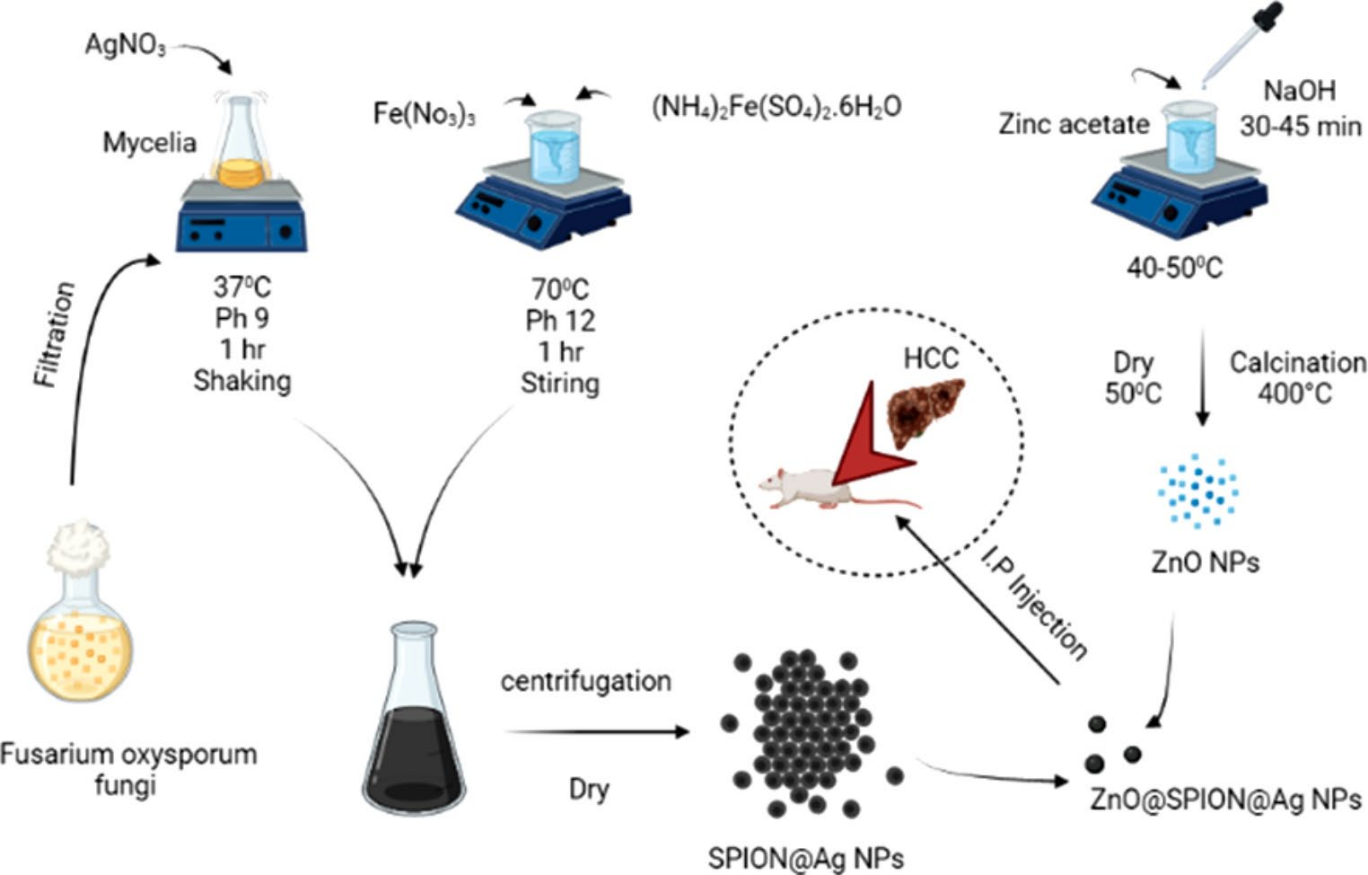
Green synthesis of ZnO@SPION@Ag nanocomposite for traetment of heptocellular carcinoma (HCC).

Drug distribution to specified sites using nanoparticles leads to its deposition in the morbid region with an appropriate drug dose and fewer side effects. Applications for iron nanoparticles or gold shells in the therapy of cancer are significant. A targeted drug decreases drug use and medical costs, making patient care more affordable. By using lipid- and polymer-based nanoparticles in the design of drug delivery systems, it is possible to enhance the therapeutic and pharmacological characteristics of drugs [75]. The capacity of drug delivery systems to change the pharmacokinetics and biodistribution of the drug is one of their strongest points. The purpose of nanoparticles is to circumvent the body’s defenses [76] and is capable of enhancing drug distribution. In order to increase efficiency, more sophisticated drug delivery systems that can pass through cell membranes and enter cell cytoplasm are being developed. Drugs that are ingested only work when they receive a certain signal. Utilizing a drug delivery system increases the drug’s solubility due to the presence of both hydrophilic and hydrophobic environments [77]. Drug distribution controls drug release to prevent tissue injury. The greater clearance of the drug from the body can be reduced by changing its pharmacokinetics. Superparamagnetic iron oxides (SPIONs) can change their surfaces to increase their colloidal stability in aqueous conditions. Additionally, it needs to offer functional groups that may be altered further to regulate drug release or bind targeted units. Nano systems based on silver nanoparticles were assessed as appropriate transporters of several therapeutic compounds, including anti-inflammatory, antioxidant, antibacterial, and anticancer chemicals. The Core-shell nanoparticles received a lot of interest, particularly since they can be used in several medicinal applications. Rashwan et al. [7] created a zinc oxide@superparamagnetic iron oxide@silver (ZnO@SPION@Ag) nanocomposite that was authorized to have an anticancer impact on hepatocellular carcinoma as represented in Fig. 7.

Due to the presence of SPION and the ability of nanocomposite to target drugs to particular cancer sites, sorafenib drug delivery systems utilizing nanocomposite are anticipated to reduce resistance and utilize low doses of the drug. Since research that was allowed showed that ZnO NPs and sorafenib worked together to treat cancer more safely and effectively while having less of an adverse effect on normal cells. Sorafenib and ZnO NPs may be coupled in novel therapeutic approaches for the treatment of cancer [78].

**Conclusion**.

The application of nanotechnology in medicine can affect human health significantly. Nanomedicine is one of the most important therapeutic techniques for multiple diseases, particularly cancer. This implies that nanomedicines are an emerging discipline that will provide an alternative to conventional therapies and therapeutic agents for effectively treating various diseases, including cancer. In this review we discussed recent advances of nanocarriers in cancer drug delivery. Recent developments in hepatocellular carcinoma (HCC) nanomedicine have yielded an effective alternative to conventional methods of tumor targeting. However, because it is difficult to discover novel pharmacologically active molecules from natural sources, improving the potency of current compounds through nanotechnology has become a common strategy. The main purpose of this study is to demonstrate that anano-based drug delivery system can effectively improve the anti-cancer capabilities of sorafenib drug to overcome the resistance crisis. Although there are still some challenges to be addressed, such as toxicity, stability, solubility, bioavailability and controlled release, nanomedicine-based drug delivery systems could potentially revolutionize cancer therapy in the future.

### Electronic supplementary material

Below is the link to the electronic supplementary material.

Supplementary Material 1

## Data Availability

The data presented in this study are available on request from the corresponding author.
