# Correction: Green synthesis of nano-based drug delivery systems developed for hepatocellular carcinoma treatment: a review

**DOI:** 10.1007/s11033-023-08909-0

**Published:** 2023-11-10

**Authors:** Doaa S. R. Khafaga, Ahmed M. El-Khawaga, Rehab Abd Elfattah Mohammed, Heba K. Abdelhakim

**Affiliations:** 1 Department of Basic Medical Sciences, Faculty of Medicine, Galala University, Suez, 43511 Egypt; 2 Faculty of medicine for girls, Internal medicine Al Azher university, Cairo, Egypt; 3 Biochemistry Division, Faculty of Science, Cairo University, Giza, 12613 Egypt

Correction: Molecular Biology Reports

10.1007/s11033-023-08823-5

In this article, the author “Ahmed M. El-khawag” was missed to be denoted as a corresponding author. The corresponding authors of this article should have been both “Doaa S. R. Khafaga” and “Ahmed M. El-khawag”. The ORCID of the author “Ahmed M. El-Khawaga” was incorrectly provided for “Doaa S. R. Khafaga”. The correct ORCID is updated for these authors.

The original article has been corrected.

