# Supplementary material for: Green synthesis of nano-based drug delivery systems developed for hepatocellular carcinoma treatment: a review
Source: Mol Biol Rep. 2023 Oct 10;50(12):10351–64. doi: 10.1007/s11033-023-08823-5 (PMC10676320; doi:10.1007/s11033-023-08823-5)
Supplement: Supplementary file 1 — Supplementary Material 1 [file 11033_2023_8823_MOESM1_ESM.docx]

**Green synthesis of nano-based drug delivery systems developed for hepatocellular carcinoma treatment: A review.**

***Doaa S. R. Khafaga ^a,*^, Ahmed M. El-Khawaga^a,*^, Rehab Abd Elfattah Mohammed^b^, Heba K. Abdelhakim ^c^***

*^a^ Department of Basic Medical Sciences, Faculty of Medicine, Galala University, Suez 43511, Egypt.*

*^b^Assistant professor internal medicine Al Azher university,faculty of medicine for girls,Cairo, Egypt.*

*^c^ Biochemistry Division, Faculty of Science, Cairo University, Giza 12613, Egypt.*

**Corresponding authors:**

*Ahmed M. Elkhawaga, Email:* [*Ahmed.Elkhawaga@gu.edu.eg*](mailto:Ahmed.Elkhawaga@gu.edu.eg)*.*

*Doaa S. R. Khafaga, Email:* [*doaa.rashwan@gu.edu.eg*](mailto:doaa.rashwan@gu.edu.eg)*,* [*doaasayed473@yahoo.com*](mailto:doaasayed473@yahoo.com)*.*

**Table S1:** Advantages and disadvantages of different type of nanocarriers

| **Nanocarrier**  **Types** | **Example** | **Advantages** | **Disadvantages** | **Ref.** |
| --- | --- | --- | --- | --- |
| Inorganic | -Silica  -Graphene oxide  -Black phosphorus | -Non-toxic  -Hydrophilic  -Biocompatible  -Highly stable | -Poor data regarding long term toxicity and stability | [1] |
| Metallic | -Gold  -Silver | -Improve Rayleigh scattering  - Improved Raman scattering  -Improve plasma absorption  -Determine chemical information on metallic nanoscale substrate  -Applied in biological system imaging | -Instability  -Biologically toxic  -Difficulty in synthesis  -Explosion  -Impurity | [2] |
| Biopolymeric | -Polyalkyl cyanoacrylate  -poly (lactic-co-glycolic -acid) polyanhydride | -Easy preparation  -High stability  -Biocompatibility  -Biodegradability | -High cost for synthesis  -Prone to aggregation  -Opsonization in the blood stream | [3] |
| Liposomes | -Triglycerides  -Fatty acids  -Partial glycerides  -Steroids  -Waxes | -Biocompatibility  -Biodegradability  - Capability to encapsulate and deliver polar and non-polar bioactive agents  - Provides sustained release and protects entrapped drug | -Low stability  -Tends to agglomerate  -Some maybe allergic | [4, 5] |
| Polymeric micelles | -Polystyrene  -poly(ethylene glycol)  -poloxamers | -Protecting drug from environmental conditions  -Controlled drug release.  -Increasing solubility of highly lipophilic drugs  -Tunable chemical and physical properties | -Use only for lipophilic drugs  -Low drug-loading capacity  -Dependency of critical micelle concentration | [6] |
| Dendrimers | Poly (propylene amine) | -Water soluble and biocompatible  -Flexibility in conjugation chemistry  -Ability to encapsulate and deliver various types of bioactive agents | -Poor drug release profile  -Toxicity concerns  -Rapid clearance  -High cost for their synthesis | [7] |

**Table S2:** Loading of some synthetic drugs on nanocarriers and their purposes.

| **Synthetic Drugs** | **Nanocomposite** | **Purpose of use** | **Ref** |
| --- | --- | --- | --- |
| Sorafenib | Sorafenib/ Fe_2_O_3_@Ag@Cs | Hepatocellular carcinoma therapy | [8] |
| Doxorubicin | DOX/MSNs-CAIX | Anticancer therapy | [9] |
| Doxorubicin | DOX/EDT-IONPs | glioblastoma therapy | [10] |
| ciprofloxacin | CIP/ PEG–PLGA | Antibiotic | [11] |
| Minocycline | minocycline-/PLGA | Antibacterial Agent | [12] |
| 5-fluorouracil | 5-FU/PLGA-Gold | Lung cancer therapy | [13] |
| 5-fluorouracil  curcumin | 5-FU/MSNs  CRC/ MSNs | Laryngeal cancer cells therapy | [14] |
| 5-fluorouracil | 5-fluorouracil /magnetoliposome | Against colon cancer | [15] |
| Quercetin  Naringin  Ceftriaxone  Ampicillin  Amphotericin B | Quercetin/BCD-ZnO  Naringin/BCD-ZnO  Ceftriaxone/BCD-ZnO  Ampicillin/BCD-ZnO  Amphotericin B/BCD-ZnO | Antibacterial Agents | [16] |
| Doxorubicin | DOX/Se@SiO2–FA–CuS | Chemo-photothermal therapy | [17] |
| Epirubicin | EPI–SPION | Anticancer Agent | [18] |

**References**

1. Paul W, Sharma CP (2010) 8 - Inorganic nanoparticles for targeted drug delivery. In: Sharma CP (ed) Biointegration of Medical Implant Materials. Woodhead Publishing, pp 204–235

2. Harish Kumar K (2018) Metallic Nanoparticle: A Review. Biomedical Journal of Scientific & Technical Research 4:001–01

3. Sundar S, Kundu J, Kundu SC (2010) Biopolymeric nanoparticles. Science and Technology of Advanced Materials 11:014104. https://doi.org/10.1088/1468-6996/11/1/014104

4. Ratemi E, Sultana Shaik A, Al Faraj A, Halwani R (2016) Alternative approaches for the treatment of airway diseases: focus on nanoparticle medicine. Clin Exp Allergy 46:1033–1042. https://doi.org/10.1111/cea.12771

5. Ghezzi M, Pescina S, Padula C, et al (2021) Polymeric micelles in drug delivery: An insight of the techniques for their characterization and assessment in biorelevant conditions. Journal of Controlled Release 332:312–336. https://doi.org/10.1016/j.jconrel.2021.02.031

6. Kahraman E, Güngör S, Özsoy Y (2017) Potential enhancement and targeting strategies of polymeric and lipid-based nanocarriers in dermal drug delivery. Ther Deliv 8:967–985. https://doi.org/10.4155/tde-2017-0075

7. Abbasi E, Aval SF, Akbarzadeh A, et al (2014) Dendrimers: synthesis, applications, and properties. Nanoscale Res Lett 9:247. https://doi.org/10.1186/1556-276X-9-247

8. A. A. Nagy R, Mohamed MH, K. A. Elhakim H, et al (2022) Anticancer effect of Sorafenib-loaded iron oxide nanoparticles and bee venom on some genes expression in hepatocellular carcinoma. Egyptian Journal of Chemistry. https://doi.org/10.21608/ejchem.2022.138553.6104

9. Chen M, Hu J, Wang L, et al (2020) Targeted and redox-responsive drug delivery systems based on carbonic anhydrase IX-decorated mesoporous silica nanoparticles for cancer therapy. Sci Rep 10:14447. https://doi.org/10.1038/s41598-020-71071-1

10. Norouzi M, Yathindranath V, Thliveris JA, et al (2020) Doxorubicin-loaded iron oxide nanoparticles for glioblastoma therapy: a combinational approach for enhanced delivery of nanoparticles. Sci Rep 10:11292. https://doi.org/10.1038/s41598-020-68017-y

11. Watcharadulyarat N, Rattanatayarom M, Ruangsawasdi N, Patikarnmonthon N (2023) PEG–PLGA nanoparticles for encapsulating ciprofloxacin. Sci Rep 13:266. https://doi.org/10.1038/s41598-023-27500-y

12. Kashi TSJ, Eskandarion S, Esfandyari-Manesh M, et al (2012) Improved drug loading and antibacterial activity of minocycline-loaded PLGA nanoparticles prepared by solid/oil/water ion pairing method. Int J Nanomedicine 7:221–234. https://doi.org/10.2147/IJN.S27709

13. Gupta R, Vishwakarma L, Guleri SK, Kumar G (2022) 5-Fluorouracil-Impregnated PLGA Coated Gold Nanoparticles for Augmented Delivery to Lung Cancer: In Vitro Investigations. Anticancer Agents Med Chem 22:2292–2302. https://doi.org/10.2174/1871520622666211224103110

14. Wang D, Yu D, Liu X, et al (2020) Targeting laryngeal cancer cells with 5-fluorouracil and curcumin using mesoporous silica nanoparticles. Technol Cancer Res Treat 19:1533033820962114. https://doi.org/10.1177/1533033820962114

15. Clares B, Biedma-Ortiz RA, Sáez-Fernández E, et al (2013) Nano-engineering of 5-fluorouracil-loaded magnetoliposomes for combined hyperthermia and chemotherapy against colon cancer. European Journal of Pharmaceutics and Biopharmaceutics 85:329–338. https://doi.org/10.1016/j.ejpb.2013.01.028

16. Akbar N, Aslam Z, Siddiqui R, et al (2021) Zinc oxide nanoparticles conjugated with clinically-approved medicines as potential antibacterial molecules. AMB Express 11:104. https://doi.org/10.1186/s13568-021-01261-1

17. Wang Y, Liu X, Deng G, et al (2018) Se@SiO 2 –FA–CuS nanocomposites for targeted delivery of DOX and nano selenium in synergistic combination of chemo-photothermal therapy. Nanoscale 10:2866–2875. https://doi.org/10.1039/C7NR09237G

18. Rao Y, Chen W, Liang X, et al (2015) Epirubicin-Loaded Superparamagnetic Iron-Oxide Nanoparticles for Transdermal Delivery: Cancer Therapy by Circumventing the Skin Barrier. Small 11:239–247. https://doi.org/10.1002/smll.201400775
